# Literature review and expert opinion on the impact of achondroplasia on medical complications and health-related quality of life and expectations for long-term impact of vosoritide: a modified Delphi study

**DOI:** 10.1186/s13023-022-02372-z

**Published:** 2022-06-13

**Authors:** Ravi Savarirayan, Wagner Baratela, Thomas Butt, Valérie Cormier-Daire, Melita Irving, Bradley S. Miller, Klaus Mohnike, Keiichi Ozono, Ron Rosenfeld, Angelo Selicorni, Dominic Thompson, Klane K. White, Michael Wright, Svein O. Fredwall

**Affiliations:** 1 Murdoch Children’s Research Institute, and University of Melbourne, Parkville, Melbourne, VIC 3052 Australia; 2 Hospital Sirio-Libanes, São Paolo, Brazil; 3 BioMarin International Ltd, London, UK; 4 Université de Paris, Reference Center for Skeletal Dysplasia, Hôpital Necker-Enfants Malades, Paris, France; 5 Guy’s and St. Thomas’ NHS Foundation Trust, London, UK; 6 University of Minnesota Medical School, Minneapolis, MN USA; 7 Universitätskinderklinik, Otto-Von-Guericke Universität, Magdeburg, Germany; 8 Osaka University Graduate School of Medicine, Osaka, Japan; 9 Oregon Health and Science University, Portland, OR USA; 10 ASST Lariana, Como, Italy; 11 Great Ormond Street Hospital for Children, NHS Foundation Trust, London, UK; 12 University of Washington, Seattle Children’s Hospital, Seattle, WA USA; 13 Northern Genetics Service, Institute of Human Genetics, Newcastle-Upon-Tyne Hospitals, NHS Foundation Trust, Newcastle-upon-Tyne, UK; 14 TRS National Resource Centre for Rare Disorders, Sunnaas Rehabilitation Hospital, Nesodden, Norway

**Keywords:** Achondroplasia, Activities of daily living, Complications, Delphi technique, Expert opinion, Growth, Health-related quality of life, Vosoritide

## Abstract

**Background:**

Achondroplasia is associated with disproportionate short stature and significant and potentially severe medical complications. Vosoritide is the first medicine to treat the underlying cause of achondroplasia and data from phase 3 and phase 2 extension studies showed effects on growth and body proportions. However, there are currently no long-term data available on the direct impact on endpoints such as medical complications and health-related quality of life (HRQoL). This study explored the perceived impact of achondroplasia on medical complications, HRQoL, healthcare resource use and mortality, and potential modifying effects of vosoritide, based on published evidence and expert opinion. Structured expert opinion was obtained by an international modified Delphi study among 14 experts in managing achondroplasia performed on a virtual platform and consisting of an explorative phase followed by an anonymous individual rating round.

**Results:**

Overall, the panelists expect that in individuals starting long-term treatment between 2 years of age and puberty, growth velocity increases observed in the clinical trials will be maintained until final height is reached (92% agreement) and will likely result in clinically meaningful improvements in upper-to-lower body segment ratio (85%). Earlier treatment initiation will likely result in a greater final height (100%) and more likely improve proportionality (92%) than later treatment. Although current data are limited, ≥ 75% of panelists find it conceivable that the earlier long-term treatment is started, the greater the probability of a positive effect on the lifetime incidence of symptomatic spinal stenosis, kyphosis, obstructive sleep apnea, and foramen magnum stenosis. These are among the most clinically important complications of achondroplasia because of their high impact on comorbidity, mortality, and/or HRQoL. A positive effect of vosoritide on the incidence of surgeries through lifetime was considered more likely with earlier long-term treatment (90%).

**Conclusions:**

This explorative study, based on international expert opinion, provides further insight into the medical and functional impacts of achondroplasia and how these might be modified through long-term use of vosoritide. The results can be used to guide the direction and design of future research to validate the assumptions and to discuss potential treatment outcomes with disease modifying therapies with families and clinicians.

**Supplementary Information:**

The online version contains supplementary material available at 10.1186/s13023-022-02372-z.

## Background

With an incidence estimated around 1 in 20,000–30,000 live births, achondroplasia is the most common disorder associated with disproportionate growth and short stature [1]. Achondroplasia is caused by a recurrent autosomal dominant gain-of-function pathogenic variant in the fibroblast growth factor receptor 3 gene (*FGFR3*), resulting in impaired endochondral ossification. The most apparent clinical manifestations of the disorder are disproportionate short stature with shortening of the upper and lower limbs, macrocephaly with frontal bossing, and midface hypoplasia. In addition, several complications can arise throughout lifetime, including life-threatening foramen magnum stenosis in infants, symptomatic spinal stenosis, thoracolumbar kyphosis, upper airway obstruction, sleep disordered breathing, genu varum/tibial bowing, recurrent otitis media, conductive hearing loss, dental malocclusion, and cardiovascular disease [1, 2]. The clinical features of achondroplasia can in turn lead to a developmental profile that differs from the norm, impaired self-care or ability to perform activities of daily living, reduced health-related quality of life (HRQoL), socioeconomic problems (e.g., reduced work participation), and early mortality [2, 3]. The development of medical complications by age and their impact on different aspects of HRQoL are being investigated in natural history studies, such as the Lifetime Impact of Achondroplasia In Europe (LIAISE) study (clinicaltrials.gov NCT03449368) and the Achondroplasia Natural History Study (CLARITY) [4]. The medical complications of achondroplasia require lifelong multidisciplinary care, which varies with age [5, 6].

Vosoritide is the first medicine to treat the underlying cause of achondroplasia. It is a biological analogue of C-type natriuretic peptide, a potent stimulator of endochondral ossification that works through downregulation of the intracellular signaling pathway of the FGFR3 receptor [7]. Vosoritide was approved by the European Medicines Agency in August 2021 for children from the age of 2 years until growth plates are closed [8] and by the US Food and Drug Administration in November 2021 for children aged ≥ 5 years with open growth plates [9]. Results of the pivotal phase 3 clinical trial showed increases in annualized growth velocity and height Z-scores after 52 weeks of treatment with vosoritide versus placebo and good tolerability [10]. Two-year follow-up data showed sustained increases in annualized growth velocity, continued improvements in height Z-score, and improvements in upper-to-lower body segment ratio versus untreated patients, without adverse effects on bone maturation [11]. Sixty-month data from a phase 2 study (N = 35) and its extension (N = 19) reported consistent findings [7, 12].

Data from the phase 3 and phase 2 extension studies showing the effects of vosoritide on growth and body proportions beyond 60 months of treatment are not yet available (clinicaltrials.gov NCT03424018 and NCT02724228). In addition, there are no long-term data currently available on the direct impact of treatment on medical complications, physical function (activities of daily living, mobility, independence), and HRQoL. To address this knowledge gap until long-term data become available, this paper explores and discusses how achondroplasia can affect medical complications, HRQoL healthcare resource use, and mortality and how vosoritide-related increases in height and improvements in other anthropometric measures such as upper-to-lower body segment ratio may impact on medical complications, physical function and HRQoL. The resulting assumptions are based on extrapolation of existing data and structured expert opinion obtained by an international modified Delphi study.

## Methods

### Study objectives

A modified Delphi study was performed to explore the potential impact of achondroplasia on medical complications / comorbidities (i.e., clinical manifestations beyond growth impairment) and the potential modifying effects of vosoritide based on published evidence and clinical opinion and expertise, and to identify evidence gaps for future clinical research.

### Participants

The expert panel consisted of a Core Committee of two (RS and SF), and twelve additional clinicians from a wide geographic spread (Australia, Brazil, France, Germany, Italy, Japan, Norway, the UK, and the USA) and covering multiple medical specialties, including clinical genetics, pediatrics, pediatric endocrinology, pediatric neurosurgery, and pediatric orthopedics. All panelists had relevant expertise in managing achondroplasia; seven (50%) are principal investigators in vosoritide clinical trials.

### Study design

**Fig. 1:**
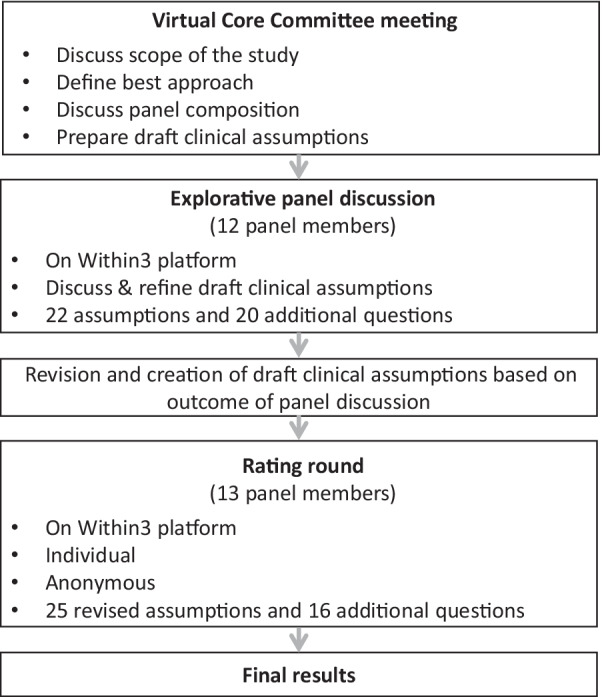
Study design of modified Delphi study

The Delphi technique is a widely used and accepted interactive research method for measuring and/or obtaining consensus of opinion from experts [13]. The technique relies on a panel of experts who anonymously answer questionnaires in two or more rounds. After each round, a summary of the experts’ feedback in the previous round is provided to the panel by a facilitator. In this study, a ‘modified’ Delphi technique was used, consisting of two main parts i.e. a structured explorative discussion phase followed by an anonymous rating round (Fig. 1).

In an initial preparatory virtual video conference meeting, the study approach, expert panel composition, and draft clinical assumptions and questions for the explorative panel discussion were discussed by the Core Committee. The subsequent explorative panel discussion was held using the Within3 platform (within3.com; a virtual engagement platform intended for healthcare professionals) to discuss and refine draft clinical assumptions and to gauge the opinion of the panel on topics for which no or limited data was available. The assumptions and questions were grouped in six major topics: anthropometrics, medical complications in general, skeletal complications, non-skeletal complications, quality of life, and mortality. For each assumption and question, a literature review of existing data was performed and summarized as background material for the panel discussion by a professional medical communication agency (Ismar Healthcare). The panelists were asked to answer questions based on published data (if available) and their own clinical experience or opinion and to explain reasons underlying their answers. Assumptions were rated on a 5-point Likert scale (strongly disagree – disagree – neutral – agree – strongly agree). Additional questions had varying answer options (Additional file 1: Tables S1–S3). Based on feedback from the expert panel, clinical assumptions were amended, and additional assumptions were formulated by Ismar Healthcare with input from the Core Committee. Finally, the revised clinical assumptions were rated individually and anonymously by the expert panel using the Within3 platform. The panelists were also asked to address additional questions on areas of interest. Percent agreement on assumptions was defined as the percentage of panelists agreeing or strongly agreeing, excluding “can’t judge” votes. BioMarin, the manufacturer of vosoritide, provided logistic support for this project, but was not involved in the voting process, or analysis of the results.

For the purpose of this paper, rating results and opinion around a selection of questions that are considered most clinically relevant, in terms of clinical impact of complications or potential mortality implications and potential impact of vosoritide, were discussed in relation to existing literature.

## Results

Twelve panel members participated in the explorative part of the study (May 24-June 13, 2021); 13 participated in the rating round (July 12–23, 2021) (Fig. 1); 11 participated in both parts of the study and 3 in one part.

Of the 22 draft assumptions discussed in the explorative phase, seven were eliminated for the anonymous rating round (Additional file 1: Table S4). Four of these, which were mainly around obesity and weight increase in individuals with achondroplasia, were rejected based on conflicting published data. Two assumptions on cardiovascular complications were eliminated because most panelists felt unable to judge them due to a lack of data or experience. One assumption was omitted for rating because it was deemed less important by the Core Committee. The remaining 15 assumptions were revised, and ten additional assumptions were formulated based on the feedback on the questions received during the explorative phase. The panel members rated the resulting 25 assumptions and responded to 16 additional questions in the rating round. Additional file 1: Tables S1–S3 provide an overview of all rating results. Expert panel agreement of ≥ 75% was obtained for 19 out of 25 assumptions; ≥ 80% agreement was obtained for 16 assumptions.

## Discussion

### Expert expectations regarding the long-term impact of vosoritide on growth velocity, height, and body proportions

Recently published follow-up data from the vosoritide phase 2 and phase 3 extension studies showed persistent improvements in annualized growth velocity in treated children with achondroplasia across age categories up to 60 months of treatment [11, 12]. Overall, the panelists of the modified Delphi study expect that growth velocity increases with vosoritide will continue beyond 60 months, until final height is reached in individuals starting treatment before puberty and that those starting treatment earlier will more likely achieve a greater final height (Table 1). It was argued that, based on the mechanism of action of vosoritide, there is no reason to believe that the gains in growth observed in the clinical trials will decelerate with continued treatment. In addition, it was noted that the pubertal period occurs over a wide age range and that the treatment response is likely to differ across this period.

**Table 1:** Assumptions and questions related to impact of vosoritide on growth velocity, height, and body proportions reaching ≥ 75% panel agreement

Assumption	% Agreement^a^	Can’t judge (%)
It is likely that long-term treatment with vosoritide increases growth velocity until final height is reached in individuals with achondroplasia starting treatment between 2 years of age and puberty (Tanner stage > 1)	92%	8
It is likely that long-term treatment with vosoritide results in a greater final height in those starting at an earlier age than in those starting later	100%	8
A clinically meaningful positive impact of vosoritide on abnormal upper-to-lower body segment ratio is more likely in individuals with achondroplasia starting long-term treatment at an earlier age than in those starting treatment later	92%	8

Question	% Likely + Very likely^b^	
How likely do you consider long-term treatment with vosoritide to result in a clinically meaningful improvement in upper-to-lower body segment ratio in individuals with ACH starting between 2 years of age and puberty (Tanner stage > 1)?	85%	0

^a^% of panel members (N = 13) agreeing or strongly agreeing with the assumption, excluding “can’t judge” votes

^b^% of panel members voting “likely” or “very likely”, excluding “Can’t judge” votes

According to 85% of the panel members, long-term treatment with vosoritide will likely or very likely result in clinically meaningful improvements in upper-to-lower body segment ratio in individuals starting treatment between 2 years of age and puberty. The panelists believe that clinical improvements in proportionality are more likely in those starting treatment earlier (100%) (Table 1). These assumptions are supported by recently published data of the phase 3 trial, that were not yet available at the time of the explorative phase of the modified Delphi study, showing statistically significant improvements in proportionality in treated patients over 2 years compared to baseline, whilst the same effect was not apparent in untreated subjects [11].

The experts emphasized that more data are needed to confirm the persistence of the effects of vosoritide on growth and body proportions for an extended period of time and the impact of age at treatment initiation on final height. These data will be provided by the ongoing open-label extensions of the phase 2 and phase 3 clinical trials. In addition, data regarding the long-term impact of vosoritide in children < 5 years of age at treatment initiation, who were excluded from the initial phase 2 and phase 3 clinical trials, are expected from two ongoing studies in young children aged 0 to 60 months (clinicaltrials.gov NCT03583697) and infants aged 0 to 12 months (clinicaltrials.gov NCT04554940) [14, 15].

### Expert expectations regarding the long-term impact of vosoritide on medical complications

**Fig. 2:**
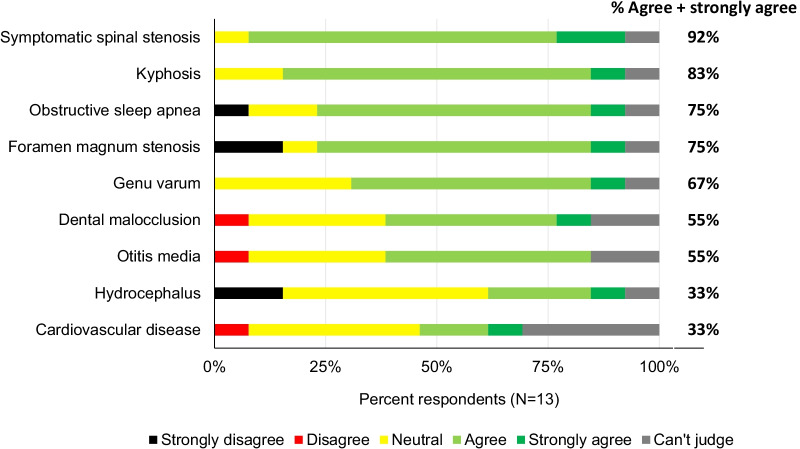
Panel rating results regarding the potential impact of vosoritide on medical complications of achondroplasia and % panel agreement. % of panel members agreeing or strongly agreeing with the assumption, excluding “can’t judge” votes. Assumption: Although current data are limited, it is conceivable that the earlier long-term treatment is started, the larger the probability of a positive impact of vosoritide on the lifetime incidence of the following medical comorbidities of achondroplasia

There is currently no available evidence for a direct effect of vosoritide on medical complications of achondroplasia. Animal studies have shown an effect of vosoritide on skull morphology (mice) and on neural foraminal area of lumbar vertebrae (cynomolgus monkeys) [16, 17], but no studies have investigated these effects in humans so far. Nevertheless, ≥ 75% of the panelists agreed or strongly agreed that it is conceivable that the earlier long-term treatment is started, the larger the probability of a positive impact of vosoritide on the lifetime incidence of symptomatic spinal stenosis, kyphosis, obstructive sleep apnea, and foramen magnum stenosis (Fig. 2). There was less agreement on other complications such as genu varum, dental malocclusion, otitis media, hydrocephalus, and cardiovascular disease. The panelists argued that, although data are limited, it seems reasonable to hypothesize, by extrapolation of the data showing the effects of vosoritide on long bone growth, that growth in the axial skeleton might be beneficially altered, resulting in a direct effect on foramen magnum stenosis, spinal canal stenosis and kyphosis. However, it was also noted that some complications such as foramen magnum stenosis may be difficult to impact due to changes that occur prenatally and in the neonatal period. A positive effect of treatment on this complication can only be expected if treatment is started very early in life. Treatment studies of infants with vosoritide are needed to verify if these early complications are targetable in infancy. The effect of treatment on foramen magnum stenosis is being investigated in an ongoing study including infants 0–12 months (clinicaltrials.gov NCT04554940) [15]. On the other hand, symptomatic spinal stenosis and kyphosis, and related back pain, are most prevalent in adults with achondroplasia and may still be altered if treatment is started at a later age, when spinal growth can still be improved. Similarly, obstructive sleep apnea can develop in infancy, but may also occur or relapse in adults with achondroplasia [18] and so might benefit from later treatment.

About one third of the panel members felt unable to judge a potential effect of treatment on cardiovascular disease, since the mechanism for increased cardiovascular mortality in patients with achondroplasia, or whether there is an increased risk at all, is not well understood. Also, the term cardiovascular disease was perceived as ‘too vague’ as it can encompass a range of different diseases. In addition, the experts noted that ‘true’ hydrocephalus, requiring neurosurgical intervention, is nowadays considered an unusual complication of achondroplasia.

### Impact of achondroplasia and potential effects of treatment on HRQoL

#### Impact of achondroplasia

It has been well established that individuals with achondroplasia have a reduced HRQoL compared with the healthy population [3, 19–21]. Several factors may contribute to this, depending on the age of the patient.

Studies have shown that short stature and skeletal dysplasia negatively impact on the ability of individuals to perform activities of daily living and on independence of adults [3, 22]. Literature suggests that in individuals with achondroplasia, limitations in physical functioning and reduced HRQoL are at least partly due to disproportionate short stature [3]. In our study, 73% of the panelists agreed that a final height < 140 cm likely has an independent negative impact on physical HRQoL (Table 2). However, it was noted that it is difficult to tease out a direct impact of short stature on HRQoL due to considerable inter-individual variability and the influence of other complications of achondroplasia beyond height. There was less agreement among the panelists regarding an independent impact of short stature on mental health (Table 2), as this may also depend on the individual’s character, age, family, and peer group. Also, there is currently limited data to support an association between short stature and reduced mental health [20, 21].

**Table 2:** Assumptions related to health-related quality of life (HRQoL) and % panel agreement

Assumption	% Agreement^a^ (%)	Can’t judge (%)
Apart from the impact of comorbidities, short stature (final height < 140 cm) likely has an independent negative impact in individuals with achondroplasia on:		
Physical health-related quality of life	73	8
Mental health-related quality of life	64	8
In individuals with achondroplasia, vosoritide likely increases HRQoL through lifetime if long-term treatment is started before puberty (Tanner stage > 1)	82	8
A positive impact of vosoritide on HRQoL is more likely in individuals with achondroplasia starting long-term treatment at an earlier age than in those starting treatment later	100	0
Increased frequency of surgeries relative to the general population has a substantial negative short-term impact on HRQoL in individuals with achondroplasia	100	0
A positive impact of vosoritide on the incidence of surgeries is more likely in individuals with achondroplasia starting long-term treatment at an earlier age than in those starting later	90	17
A positive impact of vosoritide on chronic pain through lifetime is more likely in individuals with achondroplasia starting long-term treatment at an earlier age than in those starting later	88	33
A positive impact of vosoritide on work participation through lifetime is more likely in individuals with achondroplasia starting long-term treatment at an earlier age than in those starting later	78	25
A positive impact of vosoritide on activities of daily living through lifetime is more likely in individuals with achondroplasia starting long-term treatment at an earlier age than in those starting later	73	8

^a^% of panel members (N = 12) agreeing or strongly agreeing with the assumption, excluding “can’t judge” votes

In addition to disproportionate short stature, medical complications may impact on the HRQoL of individuals with achondroplasia. Complications may result in frequent surgeries such as ear-nose-throat (mainly in children) and orthopedic procedures [4, 23], chronic pain, reduced work participation (e.g. due to symptomatic spinal stenosis in adults) and, in some cases, reduced life expectancy [19, 24]. Published data showing an impact of specific complications or surgeries on HRQoL in individuals with achondroplasia are lacking. However, based on their clinical experience, all panel members in our study agreed that an increased frequency of surgeries has a substantial negative short-term impact on HRQoL in these individuals (Table 2). Most panelists also believe that complications can still have considerable impact on HRQoL and healthcare resources even if no surgery is required (Additional file 1: Table S2). There was overall agreement that in patients with symptomatic spinal stenosis not requiring surgery, HRQoL is often affected by pain related to neurogenic claudication and limitations in the ability to perform activities of daily living (Additional file 1: Table S1). Most panelists estimated the healthcare resources for these patients for monitoring, physical and occupational therapy, assistive devices and dietary therapy to be moderate or high. These results are in accordance with findings from a Norwegian study that showed that spinal stenosis in adults with achondroplasia is often associated with pain and reduced work participation [24]. The fact that most surgeons have a high threshold for offering surgery to these patients due to the risk of suboptimal outcomes may explain the high burden of symptomatic spinal stenosis in the achondroplasia population. For individuals with kyphosis not requiring spinal fusion, most panel members estimated the impact on mobility, self-care, usual activities, and pain to be moderate or high. It was noted that kyphosis is a risk factor for spinal stenosis and that the magnitude of the impact on HRQoL is likely age-related.

The impact of foramen magnum stenosis not requiring decompression surgery on healthcare resources was considered high due to the need for monitoring and non-surgical management. In addition, 54% of panel members believe that obstructive sleep apnea can still have an impact on HRQoL when it is considered resolved after treatment, due to the recurrence risk later in life and the need for ongoing surveillance (Additional file 1: Table S1).

More data regarding the impact of achondroplasia on different domains of HRQoL, age-specific surgical burden, and the relationship between height and specific medical complications is expected from the LIAISE study, a multinational, observational, retrospective study with a cross-sectional patient-reported outcome component. Preliminary data of LIAISE showed reduced physical and psychosocial domain scores across several questionnaires and a high surgical burden in individuals with achondroplasia [23, 25]. The data also suggested a positive correlation between HRQoL and height Z-score [25].

Achondroplasia can also have an impact on mortality [2]. In the modified Delphi study, the expert panel agreed that children with achondroplasia < 5 years old have a higher or much higher risk of mortality due foramen magnum stenosis than the general population (100%) (Additional file 1: Table S3), confirming published data [1, 26, 27]. In addition, there was overall agreement that individuals with achondroplasia have a higher or much higher risk of mortality secondary to obstructive sleep apnea versus the general population (Additional file 1: Table S3). It was argued that obstructive sleep apnea is more prevalent and tends to affect individuals with achondroplasia at a younger age than in the general population. Furthermore, the respiratory sleep disturbance associated with achondroplasia, particularly in young children, is considered more complex due to the frequent combination of central and obstructive components. Although there are currently no published comparative data to confirm increased mortality in individuals with achondroplasia due to obstructive sleep apnea, studies have shown a very high prevalence of obstructive sleep apnea in children (often 50% or higher) and adults (59%) with the disorder, compared to 1–6% in the general population [18, 28].

#### Expectations regarding the direct and indirect impact of vosoritide on HRQoL

Data showing long-term effects of vosoritide on HRQoL are not yet available. However, considering the reported negative effect of severe short stature on HRQoL [3, 20], it is likely that an increase in final height may result in a better HRQoL. Increased height and longer arms may also reduce some of the limitations in mobility, activities of daily living, and independence that have been reported for adults with achondroplasia [3, 24]. Furthermore, improvements in growth may reduce the need for surgical lengthening of lower and upper limbs, which together can take several years to complete [29, 30] and may have a substantial negative impact on the patient’s HRQoL during that period.

Vosoritide may also improve HRQoL through a positive effect on particular medical complications of achondroplasia. The complications that were, according to the expert panel, most likely to improve with early vosoritide treatment are symptomatic spinal stenosis, kyphosis, foramen magnum stenosis and obstructive sleep apnea (Fig. 2). These are among the most clinically important complications of achondroplasia because of their impact on comorbidity, HRQoL, and/or mortality, as confirmed by literature [1, 4, 18, 19, 24, 26, 28, 31] and responses of the panel to questions in the modified Delphi study (see “Discussion” above and Additional file 1: Tables S1–S3). Because of the clinical importance of symptomatic spinal stenosis, kyphosis, foramen magnum stenosis and obstructive sleep apnea, evaluation of the impact of vosoritide on these complications in future clinical trials should be considered. As previously mentioned, the ongoing infant study will answer the question regarding the impact of early treatment on foramen magnum stenosis [15]. It should be noted that the perceived impact of a complication on HRQoL depends on individual patient-specific factors, such as the presence or absence and severity of other complications and their wider social environment.

There was general agreement among the panelists that vosoritide will likely improve HRQoL if long-term treatment is started before puberty. A positive impact was considered more likely in individuals starting long-term treatment at an earlier age than in those starting treatment later (Table 2). Although the experts felt that it is too early to speculate about a potential impact of vosoritide on pain, work participation, activities of daily living and the frequency of surgeries, which may all influence HRQoL, 73–90% agreed that a positive impact is more likely in individuals starting treatment at an early age (Table 2). It should be noted that 33% of the panel members felt unable to judge the potential impact of treatment on chronic pain through lifetime. Symptomatic spinal stenosis, lower limb deformities, and joint abnormalities are considered the main causes of pain in individuals with achondroplasia, so an effect of vosoritide on pain will depend, in a large part, on its impact on these complications. Future studies may help to determine the long-term impact of treatment on the incidence of surgeries, activities of daily living, pain, and work participation.

### Limitations of the study

Our study is subject to some limitations inherent to its study design. Similar to other studies relying on expert opinion, the outcomes depend on the panel composition and engagement. Although the multidisciplinary expert panel covered the most important disciplines within the management of achondroplasia patients and applied a wide geographic spread, some specialties or regions may have been underrepresented in comparison with others. In addition, while some of the panel members have been involved in the vosoritide clinical trials, others were not. This may have influenced their answers. Depending on their involvement in clinical trials and their scientific background, answers of panel members may have been subject to confirmation bias, i.e. the tendency to interpret information in a way that supports a person’s prior beliefs.

Although there is currently little or no evidence to support several of the assumptions made in the study, the responses of the panel members are based on many years of experience in the disease area and can be used to guide the design of future clinical trials until more data are available. For some of the assumptions, a substantial proportion of the panelists felt unable to make a judgement based on their own expertise or existing evidence, making these assumptions less strong. This was the case for several assumptions regarding the expected impact of vosoritide on aspects of HRQoL (Table 2). We will have to wait for real-world data to assess these outcomes and how increases in growth velocity and improvements in body proportions will impact them.

Finally, the outcome of this study must be considered as time-sensitive since it combines clinical evidence and expert opinion at a certain point in time. Ongoing research and increasing knowledge may support or refute these results.

## Conclusions

The results of this explorative study, based on international expert opinion, provides further insight into the medical and functional impacts of achondroplasia and how these might be modified through long-term use of vosoritide. The responses of the panel might be used to guide the direction and design of future research to validate the assumptions that are based on extrapolation of current data. They can also be used in discussing potential outcomes of treatment with vosoritide with families and health care providers as its use becomes more prevalent. Careful and critical prospective data collection will be required before these expert assumptions can be substantiated.

## Supplementary Information

**Additional file 1: Table S1.** Panel rating results of clinical assumptions. Green: ≥ 75% of respondents agreed or strongly agreed; yellow: 50–74% of respondents agreed or strongly agreed; orange: < 50% of respondents agreed/strongly agreed. **Table S2.** Panel rating results of questions regarding the impact of complications not requiring surgery on health-related quality of life (HRQoL) and healthcare resources. **Table S3.** Rating results of additional questions. **Table S4.** Questions eliminated after the explorative phase and reason for elimination

## Data Availability

The dataset supporting the conclusions of this article is included within the article and its additional file.
