# Supplementary material for: Literature review and expert opinion on the impact of achondroplasia on medical complications and health-related quality of life and expectations for long-term impact of vosoritide: a modified Delphi study
Source: Orphanet J Rare Dis. 2022 Jun 13;17:224. doi: 10.1186/s13023-022-02372-z (PMC9195406; doi:10.1186/s13023-022-02372-z)
Supplement: Supplementary file 1 — Additional file 1: Table S1. Panel rating results of clinical assumptions. Green: ≥ 75% of respondents agreed or strongly agreed; yellow: 50–74% of respondents agreed or strongly agreed; orange: < 50% of respondents agreed/strongly agreed. Table S2. Panel rating results of questions regarding the impact of complications not requiring surgery on health-related quality of life (HRQoL) and healthcare resources. Table S3. Rating results of additional questions. Table S4. Questions eliminated after the explorative phase and reason for elimination [file 13023_2022_2372_MOESM1_ESM.docx]

**Supplementary materials**

**Table S1** Panel rating results of clinical assumptions. Green: ≥75% of respondents agreed or strongly agreed; yellow: 50-74% of respondents agreed or strongly agreed; orange: <50% of respondents agreed/strongly agreed

| **Assumption** | **N^a^** | **Strongly disagree** | **Disagree** | **Neutral** | **Agree** | **Strongly agree** | **Can’t judge** | **% agreement^b^** |
| --- | --- | --- | --- | --- | --- | --- | --- | --- |
| **Anthropometrics** | | | | | | | | |
| In prepubertal patients (Tanner stage 1) with untreated ACH, body proportions (upper-to-lower body ratio) can be estimated based on height | 12 | 0% | 17% | 42% | 42% | 0% | 0% | 42% |
| A clinically meaningful positive impact of vosoritide on abnormal upper-to-lower body segment ratio is more likely in individuals with ACH starting long-term treatment at an earlier age than in those starting treatment later | 13 | 0% | 0% | 8% | 54% | 31% | 8% | 92% |
| It is likely that long-term treatment with vosoritide increases growth velocity until final height is reached in individuals with ACH starting treatment between 2 years of age and puberty (Tanner stage >1) | 13 | 0% | 0% | 8% | 54% | 31% | 8% | 92% |
| It is likely that long-term treatment with vosoritide results in a greater final height in those starting at an earlier age than in those starting later | 13 | 0% | 0% | 0% | 54% | 38% | 8% | 100% |
| **Medical complications in general** | | | | | | | | |
| Although current data are limited, it is conceivable that the earlier long-term treatment is started, the larger the probability of a positive impact of vosoritide on the lifetime incidence of the following medical comorbidities of ACH: | 13 |  |  |  |  |  |  |  |
| Symptomatic spinal stenosis |  | 0% | 0% | 8% | 69% | 15% | 8% | 92% |
| Kyphosis |  | 0% | 0% | 15% | 69% | 8% | 8% | 83% |
| Obstructive sleep apnea |  | 8% | 0% | 15% | 62% | 8% | 8% | 75% |
| Foramen magnum stenosis |  | 15% | 0% | 8% | 62% | 8% | 8% | 75% |
| Genu varum |  | 0% | 0% | 31% | 54% | 8% | 8% | 67% |
| Dental malocclusion |  | 0% | 8% | 31% | 38% | 8% | 15% | 55% |
| Otitis media |  | 0% | 8% | 31% | 46% | 0% | 15% | 55% |
| Hydrocephalus |  | 15% | 0% | 46% | 23% | 8% | 8% | 33% |
| Cardiovascular disease |  | 0% | 8% | 38% | 15% | 8% | 31% | 33% |
| **Quality of life** | | | | | | | | |
| Apart from the impact of comorbidities, short stature (final height <140 cm) likely has an independent negative impact in individuals with ACH on: | 12 |  |  |  |  |  |  |  |
| Physical health-related quality of life |  | 8% | 0% | 17% | 50% | 17% | 8% | 73% |
| Mental health-related quality of life |  | 0% | 17% | 17% | 42% | 17% | 8% | 64% |
| In individuals with ACH, vosoritide likely increases health-related quality of life through lifetime if long-term treatment is started before puberty (Tanner stage >1) | 12 | 0% | 0% | 17% | 58% | 17% | 8% | 82% |
| A positive impact of vosoritide on health-related quality of life is more likely in individuals with ACH starting long-term treatment at an earlier age than in those starting treatment later | 12 | 0% | 0% | 0% | 67% | 33% | 0% | 100% |
| Increased frequency of surgeries relative to the general population has a substantial negative short-term impact on health-related quality of life in individuals with ACH | 12 | 0% | 0% | 0% | 67% | 33% | 0% | 100% |
| A positive impact of vosoritide on the incidence of surgeries is more likely in individuals with ACH starting long-term treatment at an earlier age than in those starting later | 12 | 0% | 8% | 0% | 58% | 17% | 17% | 90% |
| A positive impact of vosoritide on work participation through lifetime is more likely in individuals with ACH starting long-term treatment at an earlier age than in those starting later | 12 | 0% | 0% | 17% | 50% | 8% | 25% | 78% |
| A positive impact of vosoritide on chronic pain through lifetime is more likely in individuals with ACH starting long-term treatment at an earlier age than in those starting later | 12 | 0% | 0% | 8% | 50% | 8% | 33% | 88% |
| A positive impact of vosoritide on activities of daily living through lifetime is more likely in individuals with ACH starting long-term treatment at an earlier age than in those starting later | 12 | 0% | 0% | 25% | 58% | 8% | 8% | 73% |
| **Skeletal complications** | | | | | | | | |
| The mortality risk of cervicomedullary decompression surgery in individuals with ACH is very low when performed in an optimal way and setting | 12 | 0% | 8% | 0% | 67% | 17% | 8% | 91% |
| Following decompression surgery, the risk of mortality due to foramen magnum stenosis in individuals with ACH is reduced to lower than 1% | 12 | 0% | 0% | 17% | 58% | 17% | 8% | 82% |
| In individuals with ACH who do not have surgery, spinal stenosis has no impact on mortality | 12 | 8% | 17% | 25% | 33% | 0% | 17% | 40% |
| The mortality rate associated with spinal stenosis surgery is lower than 1% in individuals with ACH | 12 | 0% | 0% | 8% | 58% | 8% | 25% | 89% |
| In individuals with ACH and symptomatic spinal stenosis not requiring surgery, HRQOL is predominantly affected by pain related to neurogenic claudication, and limitations in the ability to perform activities of daily living | 13 | 0% | 0% | 0% | 46% | 46% | 8% | 100% |
| Bilateral lower limb lengthening in individuals with ACH usually requires 2 to 4 surgical procedures | 13 | 0% | 0% | 0% | 46% | 31% | 23% | 100% |
| Bilateral upper limb lengthening usually requires 2 surgical procedures | 13 | 0% | 0% | 8% | 54% | 15% | 23% | 90% |
| **Non-skeletal complications** | | | | | | | | |
| In individuals with ACH, hydrocephalus not requiring shunting has a minimal impact on health-related quality of life | 13 | 0% | 15% | 8% | 54% | 23% | 0% | 77% |
| In current practice, surgery to manage obstructive sleep apnea is usually not performed in individuals with ACH ≥18 years of age | 13 | 0% | 15% | 31% | 31% | 8% | 15% | 45% |
| Once considered resolved, obstructive sleep apnea has no longer a significant impact on health-related quality of life | 13 | 0% | 54% | 15% | 31% | 0% | 0% | 31% |
| In current practice, if adenoidectomy/tonsillectomy is considered required in a child with ACH, it is usually done within 1 year after diagnosing obstructive sleep apnea | 13 | 0% | 0% | 0% | 77% | 15% | 8% | 100% |
| If surgery for obstructive sleep apnea in a patient with ACH is performed, the outcome (resolution of symptoms or not) is usually known within 1 year following surgery | 13 | 0% | 15% | 8% | 62% | 15% | 0% | 77% |

^a^Number of responders

^b^% Agree + Strongly agree, not taking into account “Can’t judge” responders

ACH: achondroplasia

**Table S2** Panel rating results of questions regarding the impact of complications not requiring surgery on health-related quality of life (HRQoL) and healthcare resources

| **Question** | **N^a^** | Negligible | Low | Moderate | High | Very high | **Can’t judge** |
| --- | --- | --- | --- | --- | --- | --- | --- |
| In individuals with ACH, how would you rate the impact of foramen magnum stenosis not requiring decompression surgery on the following domains of HRQoL? | 13 |  |  |  |  |  |  |
| Mobility |  | 31% | 31% | 31% | 8% | 0% | 0% |
| Self-care |  | 31% | 31% | 31% | 8% | 0% | 0% |
| Usual activities |  | 31% | 31% | 31% | 8% | 0% | 0% |
| Pain/discomfort |  | 38% | 23% | 23% | 15% | 0% | 0% |
| Anxiety/depression |  | 38% | 15% | 23% | 0% | 0% | 23% |
| Individuals with ACH who have foramen magnum stenosis not requiring decompression surgery, may need other types of care. How would you rate the impact on healthcare resources of the following: | 13 |  |  |  |  |  |  |
| Clinical monitoring |  | 0% | 0% | 46% | 38% | 15% | 0% |
| Radiological monitoring |  | 8% | 15% | 38% | 31% | 8% | 0% |
| Non-operative management (e.g., of respiratory problems) |  | 0% | 0% | 69% | 31% | 0% | 0% |
| Individuals with ACH who have symptomatic spinal stenosis not requiring surgery, may need other types of care. How would you rate the impact on healthcare resources of the following: | 13 |  |  |  |  |  |  |
| Clinical monitoring |  | 0% | 0% | 54% | 46% | 0% | 0% |
| Radiological monitoring |  | 8% | 8% | 69% | 15% | 0% | 0% |
| Physical therapy |  | 0% | 8% | 46% | 31% | 15% | 0% |
| Occupational therapy |  | 8% | 0% | 62% | 31% | 0% | 0% |
| Assistive devices |  | 0% | 0% | 54% | 38% | 0% | 8% |
| Dietary therapy (weight loss) |  | 0% | 15% | 31% | 54% | 0% | 0% |
| Others^b^ |  | 0% | 8% | 38% | 0% | 0% | 54% |
| In individuals with ACH, how would you rate the impact of kyphosis not requiring spinal fusion on the following domains of HRQoL? | 13 |  |  |  |  |  |  |
| Mobility |  | 15% | 8% | 38% | 31% | 8% | 0% |
| Self-care |  | 15% | 8% | 62% | 15% | 0% | 0% |
| Usual activities |  | 15% | 8% | 54% | 23% | 0% | 0% |
| Pain/discomfort |  | 8% | 15% | 31% | 46% | 0% | 0% |
| Anxiety/depression |  | 15% | 31% | 23% | 15% | 0% | 15% |
| Individuals with ACH who have kyphosis not requiring spinal fusion, may need other types of care. How would you rate the impact on healthcare resources of the following: | 13 |  |  |  |  |  |  |
| Clinical monitoring |  | 0% | 8% | 46% | 38% | 0% | 8% |
| Radiological monitoring |  | 0% | 8% | 54% | 23% | 8% | 8% |
| Non-operative management (e.g. bracing) |  | 8% | 8% | 54% | 23% | 0% | 8% |
| In individuals with ACH, how would you rate the impact of genu varum/ tibial bowing not requiring osteotomy on the following domains of HRQoL? | 13 |  |  |  |  |  |  |
| Mobility |  | 15% | 0% | 38% | 38% | 8% | 0% |
| Self-care |  | 15% | 38% | 46% | 0% | 0% | 0% |
| Usual activities |  | 15% | 15% | 38% | 31% | 0% | 0% |
| Pain/discomfort |  | 15% | 8% | 46% | 31% | 0% | 0% |
| Anxiety/depression |  | 15% | 38% | 38% | 0% | 0% | 8% |
| Individuals with ACH who have genu varum/tibial bowing not requiring osteotomy, may need other types of care. How would you rate the impact on healthcare resources of the following: | 13 |  |  |  |  |  |  |
| Clinical monitoring |  | 0% | 15% | 62% | 23% | 0% | 0% |
| Radiological monitoring |  | 8% | 15% | 62% | 15% | 0% | 0% |
| Non-operative management |  | 8% | 15% | 46% | 15% | 0% | 15% |
| Individuals with ACH who have hydrocephalus not requiring shunting, may need other types of care. How would you rate the impact on healthcare resources of the following: | 13 |  |  |  |  |  |  |
| Clinical monitoring |  | 0% | 8% | 46% | 31% | 8% | 8% |
| Radiological monitoring |  | 0% | 8% | 62% | 23% | 0% | 8% |
| Non-operative management |  | 8% | 31% | 38% | 8% | 0% | 15% |
| In individuals with ACH, how would you rate the impact of otitis media not requiring tympanostomy on the following domains of HRQoL? | 13 |  |  |  |  |  |  |
| Mobility |  | 46% | 46% | 8% | 0% | 0% | 0% |
| Self-care |  | 46% | 31% | 23% | 0% | 0% | 0% |
| Usual activities |  | 23% | 31% | 15% | 31% | 0% | 0% |
| Pain/discomfort |  | 8% | 23% | 46% | 23% | 0% | 0% |
| Anxiety/depression |  | 23% | 38% | 15% | 8% | 0% | 15% |
| Individuals with ACH who have otitis media not requiring tympanostomy, may need other types of care. How would you rate the impact on healthcare resources of the following: | 13 |  |  |  |  |  |  |
| Clinical monitoring |  | 8% | 15% | 46% | 31% | 0% | 0% |
| Medications |  | 15% | 15% | 54% | 15% | 0% | 0% |
| Other non-operative management |  | 8% | 46% | 15% | 31% | 0% | 0% |
| In individuals with ACH, how would you rate the impact of dental malocclusion not requiring orthodontic treatment/surgery on the following domains of HRQoL? | 12 |  |  |  |  |  |  |
| Mobility |  | 83% | 17% | 0% | 0% | 0% | 0% |
| Self-care |  | 25% | 67% | 8% | 0% | 0% | 0% |
| Usual activities |  | 25% | 42% | 33% | 0% | 0% | 0% |
| Pain/discomfort |  | 17% | 33% | 50% | 0% | 0% | 0% |
| Anxiety/depression |  | 25% | 25% | 42% | 0% | 0% | 8% |
| Individuals with ACH who have dental malocclusion and not requiring orthodontic treatment/surgery, may need other types of care. How would you rate the impact on healthcare resources of the following: | 12 |  |  |  |  |  |  |
| Clinical monitoring |  | 8% | 25% | 50% | 17% | 0% | 0% |
| Medications |  | 25% | 67% | 8% | 0% | 0% | 0% |
| Other non-operative management |  | 8% | 42% | 25% | 17% | 0% | 8% |

^a^Number of responders; ^b^Pain management, psychology

ACH: achondroplasia; CVD: cardiovascular disease; FMS: foramen magnum stenosis; OSA: obstructive sleep apnea

**Table S3** Rating results of additional questions

| **Question** | **N^a^** |  |  |  |  |  |  |  |  |  | **Can’t judge** |
| --- | --- | --- | --- | --- | --- | --- | --- | --- | --- | --- | --- |
| How likely do you consider long-term treatment with vosoritide to result in a clinically meaningful improvement in upper-to-lower body segment ratio in individuals with ACH starting between 2 years of age and puberty (Tanner stage >1)? | 13 | Very unlikely  0% | Unlikely  0% | Neutral  15% | Likely  62% | Very likely  23% |  |  |  |  | 0% |
| Which of the following medical comorbidities of ACH do you think have a comparable incidence across countries when using the same definitions? | 13 | FMS  92% | Genu varum  92% | Otitis media  92% | Hydro-cephalus  85% | Sympto-matic spinal stenosis  85% | Dental mal-occlusion  85% | Kyphosis  77% | OSA  69% | CVD  46% | 0% |
| Compared with the general population, the risk of mortality due foramen magnum stenosis in individuals with ACH <5 years old is: | 12 | Much higher  58% | Higher  33% | The same  0% | Lower  0% | Much lower  0% |  |  |  |  | 8% |
| Compared with the general population, the risk of mortality secondary to obstructive sleep apnea in individuals with ACH is | 12 | Much higher  8% | Higher  83% | The same  0% | Lower  0% | Much lower  0% |  |  |  |  | 8% |

ACH: achondroplasia

**Table S4** Questions eliminated after the explorative phase and reason for elimination

| **Assumption** | **Reason for elimination** |
| --- | --- |
| Weight increase in individuals with achondroplasia follows the same pattern as that of the average population | Rejected by the experts based on published data |
| In individuals with achondroplasia, obesity is defined as 30 kg/m^2^ | Rejected by the experts based published data |
| The relationship between height Z-score and health-related quality of life in the general population is applicable to patients with achondroplasia | Rejected by 11/12 experts; comparison with general population was considered an issue |
| Individuals with achondroplasia have the same risk of developing cardiovascular complications secondary to obstructive sleep apnea as the general population | Disagreement; 8/12 experts felt unable to judge due to limited data |
| In individuals with achondroplasia, the most frequent cardiovascular events are myocardial infarction and stroke | Disagreement; 9/12 experts felt unable to judge due to limited data |
| The following conditions have no impact on mortality in achondroplasia patients: limb lengthening, chronic pain, otitis media and tympanostomy | Accepted by most experts, but considered less important |
| The risk of mortality associated with obesity is similar for the average stature and achondroplasia populations | Rejected by the experts based on published data |
